# Detection of Ciprofloxacin-Resistant, *β*-Lactamase–Producing *Neisseria meningitidis* Serogroup Y Isolates — United States, 2019–2020

**DOI:** 10.15585/mmwr.mm6924a2

**Published:** 2020-06-19

**Authors:** Lucy A. McNamara, Caelin Potts, Amy E. Blain, Adam C. Retchless, Natashia Reese, Stephanie Swint, David Lonsway, Maria Karlsson, Kristy Lunquest, John J. Sweitzer, Xin Wang, Susan Hariri, LeAnne M. Fox, Antimicrobial-Resistant Neisseria meningitidis Team, Antimicrobial-Resistant Neisseria meningitidis Team, Nirmala Dhungana, Ryan Gabrio-Brannon, Jennifer Kyle, Brittany Martin, Meghan Barnes, Ashley Moore, Susan Hannagan, Page Keating, Sandy Li, Justin Albertson, Wayne Fleming, Perrianne Lurie, Christina Russell, Kara Reid, Kelsey Sanders, Chas DeBolt, Nicholas Graff, Esther Lam, Benjamin Hanisch, Gillian Taormina

**Affiliations:** ^1^Division of Bacterial Diseases, National Center for Immunization and Respiratory Diseases, CDC; ^2^Division of Healthcare Quality Promotion, National Center for Emerging and Zoonotic Infectious Diseases, CDC; ^3^Maryland Department of Health, Baltimore.; California Department of Public Health; California Department of Public Health; California Department of Public Health; California Department of Public Health; Colorado Department of Public Health and Environment; Georgia Department of Public Health; New Jersey Department of Health; New York City Department of Health and Mental Hygiene; New York City Department of Health and Mental Hygiene; North Carolina Department of Health and Human Services; Bureau of Epidemiology; Pennsylvania Department of Health; Bureau of Epidemiology; Pennsylvania Department of Health; Bureau of Laboratories; Pennsylvania Department of Health; Philadelphia Department of Health; Texas Department of State Health Services; Washington State Department of Health; Washington State Department of Health; Washington State Department of Health; Children’s National Hospital; Washington, DC; Children’s National Hospital; Washington, DC

Meningococcal disease is a sudden-onset, life-threatening illness caused by the bacterium *Neisseria meningitidis*. Prompt empiric antibiotic treatment can reduce morbidity and mortality among patients, and antibiotic prophylaxis can prevent secondary disease in close contacts. Historically, *N. meningitidis* isolates in the United States have largely been susceptible to the antibiotics recommended for treatment and prophylaxis, including penicillin and ciprofloxacin. This report describes detection of penicillin-resistant and ciprofloxacin-resistant *N. meningitidis* serogroup Y (NmY) isolates in the United States. NmY isolates containing a *bla*_ROB-1_ *β*-lactamase enzyme gene conferring resistance to penicillins (*1*) were recovered from 33 cases reported during 2013–2020. Isolates from 11 of these cases, reported during 2019–2020, harbored a ciprofloxacin resistance–associated mutation in a chromosomal gene (*gyrA*). Cases were reported from 12 geographically disparate states; a majority of cases (22 of 33, 67%) occurred in Hispanic persons. These cases represent a substantial increase in penicillin-resistant and ciprofloxacin-resistant meningococci in the United States since 2013. Ceftriaxone and cefotaxime, the recommended first-line agents for empiric bacterial meningitis treatment, can continue to be used for treatment, but health care providers should ascertain susceptibility of meningococcal isolates to penicillin before switching to penicillin or ampicillin. Ongoing monitoring for antimicrobial resistance among meningococcal isolates and prophylaxis failures will be important to inform treatment and prophylaxis recommendations.

Meningococcal disease is a severe illness with a sudden onset and 10%–15% case-fatality rate. The disease is typically treated empirically with cefotaxime or ceftriaxone, which can be changed to penicillin or ampicillin once *N. meningitidis* is confirmed as the causative pathogen (*2*). Because close contacts of meningococcal disease patients have an elevated risk for disease (*3*), they are recommended to receive antibiotic prophylaxis with ciprofloxacin, rifampin, or ceftriaxone as soon as a suspected meningococcal disease case is identified (*4*).

Resistance to the antibiotics used for meningococcal treatment and prophylaxis has been rare among *N. meningitidis* isolates in the United States (*5*). Although intermediate penicillin susceptibility is common among meningococci, the clinical relevance of this finding is unclear. Penicillin resistance in *N. meningitidis* attributable either to *β*-lactamase production or to other mechanisms is rare (*5*,*6*). Resistance to ciprofloxacin is also uncommon in the United States with only one identified cluster of three ciprofloxacin-resistant cases during 2007–2008 and infrequent sporadic cases (*5*,*7*,*8*). Because *N. meningitidis* is typically susceptible to clinically relevant antibiotics in the United States, antimicrobial susceptibility testing is not routinely performed on meningococcal isolates (*9*).

In January 2020, an NmY isolate that produced a *β*-lactamase and was resistant to penicillin and ciprofloxacin was cultured from a meningococcal disease case in a Maryland resident (Gillian Taormina, Benjamin Hanisch, Children’s National Hospital, Washington, DC, personal communication; 2020). When a second case of infection with a *β*-lactamase–producing, ciprofloxacin-resistant NmY isolate was reported by the Maryland Department of Health in February 2020, a systematic analysis of *N. meningitidis* isolates in the United States was conducted to determine whether this resistance pattern was more widespread.

Isolates from meningococcal disease cases are submitted to CDC approximately every 6 months by health departments from all states, Washington, D.C., and New York City. For this investigation, CDC requested that health departments submit to CDC all NmY isolates from cases during 2019–2020 that had not yet been submitted. The request was made through CDC’s Epi-X (https://emergency.cdc.gov/epix/index.asp) secure communications network for public health officials with follow-up by e-mail to each state health department. Isolates, or confirmation that no additional isolates were available, were received from 24 state health departments and the District of Columbia.

Whole genome sequencing (WGS) was performed on all available meningococcal isolates from U.S. invasive meningococcal disease cases that occurred during 2011–2020. Sequencing data were analyzed to assess the presence of the *bla*_ROB-1_ *β*-lactamase gene and mutations associated with ciprofloxacin resistance. Isolates with both a *β*-lactamase gene and ciprofloxacin resistance–associated mutations underwent reference antimicrobial susceptibility testing at CDC to assess *β*-lactamase activity and susceptibility to penicillin, ciprofloxacin, and third-generation cephalosporins. State health departments provided supplementary epidemiologic data from case investigation records for cases with isolates containing a *β*-lactamase gene.

A total of 2,097 *N. meningitidis* isolates underwent WGS; 372 of these isolates were NmY. Analysis of WGS data identified 11 serogroup Y isolates that contained a *bla*_ROB-1_ *β*-lactamase gene and a T91I *gyrA* mutation associated with resistance to ciprofloxacin. An additional 22 isolates contained *bla*_ROB-1_ but did not have mutations associated with ciprofloxacin resistance; 21 of these isolates were serogroup Y while one was nongroupable (NG). All 33 *β*-lactamase–containing isolates were in clonal complex 23 (CC23); 30, including all 11 with ciprofloxacin resistance mutations, were sequence type (ST)-3587; two were ST-15379; and one was ST-13034. The 33 isolates were from cases occurring in 12 states during 2013–2020 (Figure 1) (Figure 2). Antimicrobial susceptibility testing was conducted on the 11 isolates with ciprofloxacin resistance mutations; all were confirmed to produce a *β*-lactamase and to be resistant to penicillin and ciprofloxacin but susceptible to third-generation cephalosporins, rifampin, and azithromycin.

**FIGURE 1:**
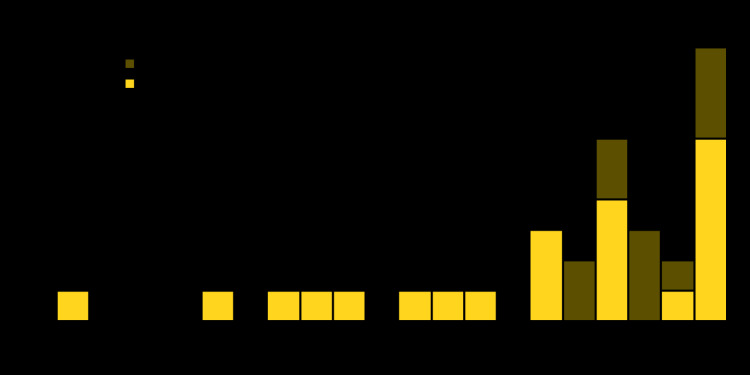
Clonal complex 23 Neisseria meningitidis isolates (N = 33) with a *bla*ROB-1 *β*-lactamase enzyme gene* alone or in combination with a ciprofloxacin resistance–associated mutation (cipro-R), by quarter — United States, 2013–2020 * Conferring resistance to penicillins.

**FIGURE 2:**
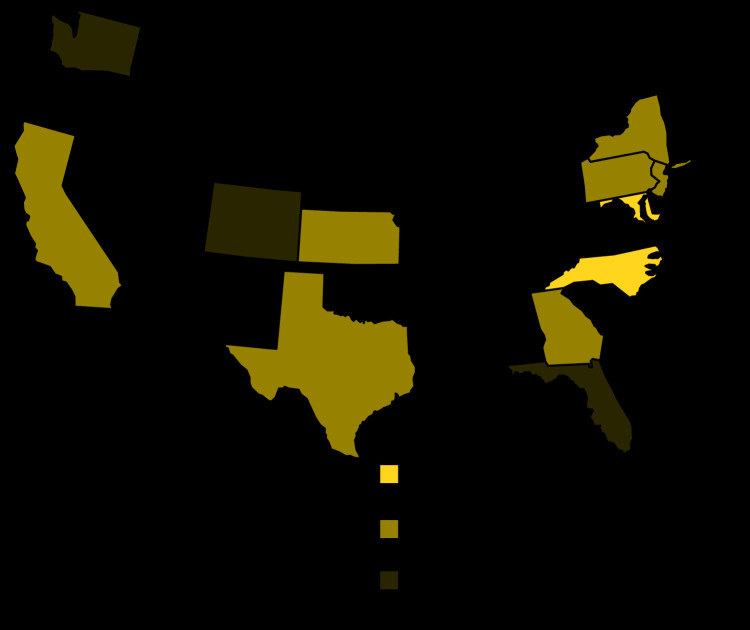
Meningococcal disease cases with clonal complex 23 Neisseria meningitidis isolates (N = 33) with a *bla*ROB-1 *β*-lactamase enzyme gene* alone or in combination with a ciprofloxacin resistance–associated mutation, by state — United States, 2013–2020 Abbreviation: DC = District of Columbia. * Conferring resistance to penicillins.

A majority of the meningococcal disease cases caused by isolates containing *bla*_ROB-1_ occurred in young children and older adults (Table). Notably, although there were no known epidemiologic links among the 33 cases, 22 (67%) occurred in Hispanic persons, including eight of the 11 cases with ciprofloxacin-resistant isolates. Only one case was fatal (case-fatality rate = 3.0%).

**TABLE:** Epidemiologic and clinical characteristics of meningococcal disease cases caused by *bla*_ROB-1_-containing *Neisseria meningitidis*, United States, 2013–2020

Characteristic	No. (%)		
	All ROB-1+*	ROB-1+ only	ROB-1+, ciprofloxacin-resistant
**Total**	**33**	**22**	**11**
**Age group (yrs)**			
<1	6 (18)	3 (14)	3 (27)
1–10	4 (12)	3 (14)	1 (9)
11–23	2 (6.1)	1 (4.5)	1 (9)
24–44	6 (18)	4 (18)	2 (18)
45–64	10 (30)	7 (32)	3 (27)
≥65	5 (15)	4 (18)	1 (9)
**Sex**			
Male	18 (54)	9 (41)	9 (82)
Female	15 (45)	13 (59)	2 (18)
**Race/Ethnicity**			
Hispanic	22 (67)	14 (64)	8 (73)
White, Non-Hispanic	4 (12)	4 (18)	0 (—)
Black^†^	6 (18)	3 (14)	3 (27)
Unknown	1 (3.0)	1 (4.5)	0 (—)
**Outcome**			
Survived	32 (97)	21 (95)	11 (100)
Died	1 (3.0)	1 (4.5)	0 (—)

* ROB-1+ is a *β*-lactamase enzyme gene that confers resistance to penicillins.

^†^ Ethnicity of two black patients was not reported. Remaining black patients were non-Hispanic.

## Discussion

This evaluation identified a novel, emerging strain of penicillin-resistant and ciprofloxacin-resistant, *β*-lactamase–producing *N. meningitidis* in the United States. The detection of geographically diverse cases with penicillin-resistant and ciprofloxacin-resistant NmY isolates has implications for treatment and prophylaxis of meningococcal disease in the United States.

Ceftriaxone and cefotaxime are the recommended first-line agents for empiric bacterial meningitis treatment and can continue to be used (*2*). However, given the number of *β*-lactamase–producing isolates detected and availability of other effective treatment options, health care providers in the United States should ascertain susceptibility of meningococcal isolates to penicillin before using penicillin or ampicillin for treatment. 

Ongoing monitoring for antimicrobial resistance among meningococcal isolates and for prophylaxis failures will be important to inform whether changes to meningococcal disease prophylaxis guidance is needed. A 2-day course of rifampin or a single injection of ceftriaxone are recommended alternatives to ciprofloxacin for prophylaxis of contacts of persons with meningococcal disease (*4*) but are logistically more challenging to administer. A single dose of azithromycin can also be used for prophylaxis in communities where ciprofloxacin-resistant meningococci have been detected; however, there is only a single published study demonstrating effectiveness of azithromycin for clearing meningococcal carriage (*4*).

It is unknown how widely the *β*-lactamase–positive, ciprofloxacin-resistant NmY strain detected in the United States might be circulating in other countries. Penicillin-resistant and ciprofloxacin-resistant NmY isolates were detected in El Salvador during 2017–2019, but similar cases have not been reported elsewhere. Single NmY isolates positive for *bla*_ROB-1_ *β*-lactamase but susceptible to ciprofloxacin have also been reported from Canada (*6*) and France (*10*). The potential circulation of penicillin-resistant or ciprofloxacin-resistant meningococci in other countries merits further investigation.

These findings show that penicillin-resistant and ciprofloxacin-resistant meningococci are now present in the United States; however, the complete geographic and temporal distribution of these resistant meningococci is unclear, because not all U.S. meningococcal disease cases have isolates available for WGS or antimicrobial susceptibility testing. In 2017 and 2018, CDC received isolates for only 72% and 78% of U.S. meningococcal disease cases, respectively*; submission of isolates from meningococcal disease cases that occurred during 2019–2020 is ongoing. The coronavirus disease 2019 (COVID-19) pandemic has limited the submission of meningococcal isolates and collection of epidemiologic data and precluded phenotypic antimicrobial susceptibility testing on all isolates containing a *β*-lactamase gene.

To facilitate ongoing monitoring of antimicrobial resistance, state and territorial health departments are asked to continue submitting all meningococcal isolates to CDC for antimicrobial susceptibility testing and WGS and to report any suspected meningococcal treatment or prophylaxis failures. In states that have experienced meningococcal disease cases caused by ciprofloxacin-resistant strains during the past 1–2 years, clinicians and public health staff members should consider antimicrobial susceptibility testing on meningococcal isolates to inform prophylaxis decisions.^†^ Antimicrobial susceptibility testing should not delay the initiation of prophylaxis. Jurisdictions with capacity for *β*-lactamase screening or WGS might also wish to assess *β*-lactamase production or presence of *β*-lactamase genes and ciprofloxacin resistance-associated mutations. States conducting their own antimicrobial susceptibility testing, *β*-lactamase screening, or WGS are requested to share results and sequences with CDC. For cases with isolates determined to be *β*-lactamase screen-positive or ciprofloxacin-resistant, jurisdictions are requested to obtain and submit a supplementary case report form (https://www.cdc.gov/meningococcal/surveillance/index.html).

**Summary**

**What is already known about this topic?**

Most *Neisseria meningitidis* isolates in the United States have been susceptible to antibiotics recommended for treatment and prophylaxis.

**What is added by this report?**

During 2019–2020, 11 meningococcal isolates from U.S. patients had isolates containing a *bla*_ROB-1_ *β*-lactamase gene associated with penicillin resistance and mutations associated with ciprofloxacin resistance. An additional 22 cases reported during 2013–2020 contained *bla*_ROB-1_ but did not have mutations associated with ciprofloxacin resistance.

**What are the implications for public health practice?**

Ceftriaxone and cefotaxime can continue to be used for empiric bacterial meningitis treatment; meningococcal isolate susceptibility to penicillin should be determined before switching to penicillin or ampicillin. Prophylaxis failures and antimicrobial resistance among meningococcal isolates should be monitored to inform meningococcal prophylaxis recommendations.

## Footnotes

* https://www.cdc.gov/meningococcal/downloads/NCIRD-EMS-Report-2017.pdf and https://www.cdc.gov/meningococcal/downloads/NCIRD-EMS-Report-2018.pdf.

† Rigorous protection from droplets and aerosols, including use of a biosafety cabinet, is required when microbiologic procedures are performed on *N. meningitidis* isolates (https://www.cdc.gov/meningitis/lab-manual/chpt11-antimicrobial-suscept-testing.html). Microbiologists who are routinely exposed to *N. meningitidis* isolates should receive meningococcal vaccines in accordance with current Advisory Committee on Immunization Practices recommendations (https://www.cdc.gov/vaccines/hcp/acip-recs/vacc-specific/mening.html).
